# Effects of SOX2 on Proliferation, Migration and Adhesion of Human Dental Pulp Stem Cells

**DOI:** 10.1371/journal.pone.0141346

**Published:** 2015-10-23

**Authors:** Pengfei Liu, Jinglei Cai, Delu Dong, Yaoyu Chen, Xiaobo Liu, Yi Wang, Yulai Zhou

**Affiliations:** 1 Department of Regenerative Medicine, School of Pharmaceutical Science, Jilin University, Changchun, P.R. China; 2 Key Laboratory of Regenerative Biology, Guangdong Provincial Key Laboratory of Stem Cell and Regenerative Medicine, Guangzhou Institutes of Biomedicine and Health, Chinese Academy of Sciences, Guangzhou, P.R. China; Second University of Naples, ITALY

## Abstract

As a key factor for cell pluripotent and self-renewing phenotypes, SOX2 has attracted scientists’ attention gradually in recent years. However, its exact effects in dental pulp stem cells (DPSCs) are still unclear. In this study, we mainly investigated whether SOX2 could affect some biological functions of DPSCs. DPSCs were isolated from the dental pulp of human impacted third molar. SOX2 overexpressing DPSCs (DPSCs-SOX2) were established through retroviral infection. The effect of SOX2 on cell proliferation, migration and adhesion ability was evaluated with CCK-8, trans-well system and fibronectin-induced cell attachment experiment respectively. Whole genome expression of DPSCs-SOX2 was analyzed with RNA microarray. Furthermore, a rescue experiment was performed with SOX2-siRNA in DPSC-SOX2 to confirm the effect of SOX2 overexpression in DPSCs. We found that SOX2 overexpression could result in the enhancement of cell proliferation, migration, and adhesion in DPSCs obviously. RNA microarray analysis indicated that some key genes in the signal pathways associated with cell cycle, migration and adhesion were upregulated in different degree, and the results were further confirmed with qPCR and western-blot. Finally, DPSC-SOX2 transfected with SOX2-siRNA showed a decrease of cell proliferation, migration and adhesion ability, which further confirmed the biological effect of SOX2 in human DPSCs. This study indicated that SOX2 could improve the cell proliferation, migration and adhesion ability of DPSCs through regulating gene expression about cell cycle, migration and adhesion, and provided a novel strategy to develop seed cells with strong proliferation, migration and adhesion ability for tissue engineering.

## Introduction

In the field of regenerative medicine, stem cells have been investigated for years, for their ability to repair injured tissue and restore organ function partially. Many kinds of stem cells have been applied in disease treatment, even in clinic [1, 2]. However, compared with embryonic stem cells, more kinds of adult stem cells have been used in clinical disease treatment without any ethical controversy [2, 3]. Moreover, adult stem cells have been identified in lots of organs and tissues, including bone marrow, peripheral blood, skin, tooth, and so on. Among these organs or tissues, dental pulp is considered as an interesting source of adult stem cells, due to the low-invasive procedures required for cell isolation and the high content of cells, compared with other adult tissue sources [4–6]. Dental pulp stem cells (DPSCs) are a class of adult stem cells present in the dental pulp, holding the potential of self-renewal and multilineage differentiation [7–9]. Even though few clinical trials about DPSCs have been reported so far, lots of scientists have highlighted that DPSCs indeed hold the potential to be used in clinical studies and disease treatments [10–13]. Unfortunately, the proliferation ability and lifespan of DPSCs are limited, which further limit the application of DPSCs in regenerative medicine. In addition, their stem cell properties are lost gradually during the expansion process in vitro [14–17]. Liu et al. investigated the expression of OCT4, SOX2, and cMYC in the human DPSCs cultured in vitro at various passages, and demonstrated that the expression of those genes was detectable in the early passaged DPSCs, and down-regulated gradually with further passage [17]. Therefore, the current culture conditions might be not fit for the maintenance of stemness of human DPSCs during the long-term in vitro cultivation.

In recent years, to induce pluripotent stem cells and enhance the stemness of somatic cells and adult stem cells, forced expression of pluripotent cell-specific factors (OCT4, SOX2, KLF4 and cMYC) has been shown to induce somatic cells and adult stem cells reprogramming into induced pluripotent stem cells (iPS cells) successfully [18–21]. Scientists also have reprogrammed DPSCs into iPS cells, and demonstrated the promising potential of DPSC collection as a source of iPS cell banks for application in regenerative medicine [22–24]. Among the four pluripotent factors, SOX2 holds the potential to maintain the characteristics of embryonic and neural stem cells, which is considered as a member of the SRY-related HMG box family, and it is essential to pluripotent and self-renewing phenotypes [16, 25]. Moreover, some novel functions of SOX2 on the regulation of cell differentiation also have been demonstrated gradually. Some researchers have indicated that the overexpression of SOX2 could promote embryonic stem cells to differentiate into cells expressing some markers for mesoderm, neuroectoderm, and trophectoderm cells [16, 26]. Han et al. found that the OCT4/SOX2-overexpressing adipose tissue-derived mesenchymal stem cells showed an improvement in cell proliferation and differentiation abilities for adipocytes or osteoblasts compared with the normal stem cells [16]. Recently, Qin et al established a method to convert adipose tissue-derived mesenchymal stem cells into induced neural stem cells with a single transcription factor SOX2, providing another alternative cell source for cell therapy of neurological disorders [27]. However, some studies have indicated that the expression of SOX2 is usually at low levels in early-passage mesenchymal stem cells and down-regulated gradually with cell passage [16, 28, 29], and the exact effects of SOX2 in DPSCs are still unclear, even though several researchers have studied the effect of SOX2 overexpression in some kinds of cells [28, 30, 31].

Recently, the functions of another stem cell factor, OCT4A, were explored in human DPSCs by scientists [14]. Similar to SOX2, OCT4A is also mainly expressed in the nucleus of stem cells and regulate the maintenance of pluripotency and self-renewal. The scientists’ research indicated that OCT4A-overexpressed DPSCs showed an up-regulation of OCT4A, OCT4B1, NANOG, SOX2, KLF4, cMYC, and UTF1. Besides, the cell proliferation ability was also significantly enhanced, as well as odontogenic and adipogenic differentiation ability [14]. Therefore, their study demonstrated that OCT4A hold a critical role to regulate cell proliferation, pluripotency, and multilineage differentiation potential of human DPSCs, and OCT4A-overexpressed DPSCs might hold special advantage in tissue engineering, used as a novel kind of seed cells. However, whether overexpression of SOX2 could still improve the proliferation ability and limited lifespan of DPSCs is still far away from our understanding.

In the present study, human SOX2 gene was introduced into human DPSCs, and its overexpression was evaluated to determine the biological effects of SOX2 in DPSCs, as well as whether SOX2 could enhance the proliferation ability and other biological function of DPSCs.

## Materials and Methods

This study was approved by the Committee on the Human Subject Research Ethics Committee of School of Pharmaceutical Science, Jilin University.

### Isolation and Characterization of Human DPSCs

Normal human impacted third molar was collected from patient after giving informed consent. The participant has provided the written informed consent to participate in this study. DPSCs were isolated using the method described as the reference [32]. Briefly, after cleaning the tooth surface and cutting around the cementoenamel junction, the dental pulp was isolated and further digested in a solution of 3 mg/mL collagenase type I for 1 hour at 37°C. Single-cell suspensions were obtained by passing the digested tissues through a 70-mm cell strainer (BD). Cell suspensions of dental pulp were seeded into 6-well plate and cultured with Alpha-Minimum Essential Medium (Gibco) supplemented with 10% fetal bovine serum (FBS, Gibco), 100 U/mL penicillin, and 0.1g/mL streptomycin (Hyclone) at 37°C under 5% CO2 condition. The medium was changed every 3 days, and cells at passages 3 or 4 were used for further experiments. For the characterization, DPSCs was prepared for phenotypic analysis by flow cytometry. The cells were then screened for CD14, CD29, CD34, CD44, CD45, CD73, CD90, CD105, and CD166. Besides, DPSCs were differentiated into adipocytes and osteocytes as previously described [33, 34].

### Establishment of SOX2 Overexpressing Cell Lines

To generate SOX2 overexpressing vectors, the SOX2-coding sequences were obtained by reverse transcription PCR and cloned into pMXs-based retroviral plasmid (Addgene). Human DPSCs were infected as described [20, 21], to establish SOX2 overexpressing DPSCs (DPSCs-SOX2), and DPSCs infected with retrovirus containing blank pMXs vector (DPSCs-vector) were used as the control group in the following detections.

### Real-Time Quantitative PCR

Total RNA was extracted with Trizol (Invitrogen). Then, 2 mg of RNA was reverse transcribed using RT-PCR kit (Takara) and qPCR was performed using a Thermal Cycler Dice^TM^ Real Time System and SYBR Green Premix EX Taq^TM^ (Takara). In our study, β-actin was used for Real-time quantitative PCR (qPCR) normalization and all items were measured in triplicate. All primer sequences (5’-3’) are as follows:

CycA    forward (F) 5’-GGATGGTAGTTTTGAGTCACCAC-3’

             reverse (R) 5’-CACGAGGATAGCTCTCATACTGT-3’

CycD1   forward (F) 5’-GCTGCGAAGTGGAAACCATC-3’

             reverse (R) 5’-CCTCCTTCTGCACACATTTGAA-3’

CycE    forward (F) 5’-GCCAGCCTTGGGACAATAATG-3’

             reverse (R) 5’-CTTGCACGTTGAGTTTGGGT-3’

CDK2    forward (F) 5’-CCAGGAGTTACTTCTATGCCTGA-3’

              reverse (R) 5’-TTCATCCAGGGGAGGTACAAC-3’

Cdc25A  forward (F) 5’-TTCCTCTTTTTACACCCCAGTCA-3’

             reverse (R) 5’-TCGGTTGTCAAGGTTTGTAGTTC-3’

Cdc14A  forward (F) 5’-CGAGCACTATGACCTCTTCTTCA-3’

             reverse (R) 5’-AGGCTATCAATGTCCCTGTTCT-3’

PI3K    forward (F) 5’-AGCCCAGTGACATCAACACTT-3’

             reverse (R) 5’-CCGCATCATAGGTCGATTTCAA-3’

EDN1    forward (F) 5’-AAGGCAACAGACCGTGAAAAT-3’

             reverse (R) 5’-CGACCTGGTTTGTCTTAGGTG-3’

GRB2    forward (F) 5’-ATTCCTGCGGGACATAGAACA-3’

             reverse (R) 5’-GGTGACATAATTGCGGGGAAAC-3’

THBS1   forward (F) 5’-GCCATCCGCACTAACTACATT-3’

             reverse (R) 5’-TCCGTTGTGATAGCATAGGGG-3’

ITGB1   forward (F) 5’-CAAGAGAGCTGAAGACTATCCCA-3’

             reverse (R) 5’-TGAAGTCCGAAGTAATCCTCCT-3’

ITGA3   forward (F) 5’-CTACCACAACGAGATGTGCAA-3’

             reverse (R) 5’-CCGAAGTACACAGTGTTCTGG-3’

CLDN1   forward (F) 5’-CCTCCTGGGAGTGATAGCAAT-3’

             reverse (R) 5’-GGCAACTAAAATAGCCAGACCT-3’

CLDN2   forward (F) 5’-GCCTCTGGATGGAATGTGCC-3’

             reverse (R) 5’-GCTACCGCCACTCTGTCTTTG-3’

CSNK2B  forward (F) 5’-TGAGCAGGTCCCTCACTACC-3’

             reverse (R) 5’-GTAGCGGGCGTGGATCAAT-3’

F11R    forward (F) 5’-GTGCCTACTCGGGCTTTTCTT-3’

             reverse (R) 5’-GTCACCCGGTCCTCATAGGAA-3’

HRAS    forward (F) 5’-ATGACGGAATATAAGCTGGTGGT-3’

             reverse (R) 5’-GGCACGTCTCCCCATCAATG-3’

JAM3    forward (F) 5’-CGGCTGCCTGACTTCTTCC-3’

             reverse (R) 5’-TGGGGTTCGATTGCTGGATTT-3’

β-actin forward (F) 5’-CCCAGAGCAAGAGAGG-3’

             reverse (R) 5’-GTCCAGACGCAGGATG- 3’

### Flow Cytometry

DPSCs were dissociated into single cells with 0.25% trypsin, further fixed and permeated with Fixation Buffer (BD) and Perm/Wash Buffer (BD), respectively. Finally, the cells were prepared at a concentration of 1.0×10^5^ cells in 100 μL of PBS. The antibodies, including CD14 conjugated to FITC (BD), CD29 conjugated to FITC (BD), CD34 conjugated to PE (BD), CD44 conjugated to FITC (BD), CD45 conjugated to PE (BD), CD73 conjugated to APC(BD), CD90 conjugated to FITC (BD), CD105 conjugated to APC (BD), CD166 conjugated to PE (BD), and SOX2 conjugated to PerCP (BD), were added and incubated for 30 min at 4°C. After two washes in PBS, the cells were acquired and analyzed with FACScalibur (BD Bioscience). The software “FlowJo 7.6.1” was used to analyze gene expression herein.

### Western Blot

In our experiment, DPSCs were harvested at the indicated times with RIPA lysis buffer (50 mM Tris–HCl, pH 7.4; 1% TritonX-100; 150 mM NaCl; 1% sodium deoxycholate; 0.1% SDS), including the phosphatase inhibitors (sodium orthovanadate, 2 mM) and protease inhibitor (0.5 mg/mL leupeptin, 0.1 mg/mL aprotinin, 0.6 mg/mL pepstatin A), for 30 min. After centrifugation at 12,000 rpm for 15 min, the protein content of cell lysates was determined using a BCA protein estimation kit (Pierce, USA), and bovine serum albumin was used as the standard. Equal amounts (15 mg) of protein were loaded per lane and electrophoresed in a 12% acrylamide gel, which was run at 120 V for 1 h. Protein transfer was performed using nitrocellulose for 1 h at 100 V. The primary antibodies used were anti-CycA (1:500; Santa), anti-CycD1 (1:200; Santa), anti-PI3K (1:200; Santa), anti-EDN1 (1:400; Santa), anti-CLDN1 (1:500; Santa) and anti-CLDN2 (1:300; Santa). Anti-mouse, rabbit or goat HRP and an Amersham ECL kit (GE Healthcare) were used to detect protein. The band densities were quantified by densitometry (Quantity One v4.62).

### Cell Proliferation and Cell Cycle Analysis

For the evaluation of cell proliferation ability, the proliferation index of each group was determined using the CCK-8 method (Dojindo) according to the manufacturer’s instructions. In brief, 10 μL of CCK-8 solution was added into each well (containing 100 μL of medium), and further cultured for 1 h to 2 h at 37°C. The absorbance of each group at 450 nm was detected (n = 4) and it was directly proportional to the number of living cells. In our study, the proliferation index = the absorbance of experimental group—the absorbance of blank group, was used to measure cell proliferation ability.

To analyze cell cycle, the cells of each group were washed twice with PBS and harvested by trypsin. Then, the cells were fixed with 70% ethanol at 4°C for 12 hours. The fixed cells were washed with ice-cold PBS and stained with propidium iodide (Beyotime) in the presence of RNase A (Beyotime) for 30min as the manufacturer’s instructions. Finally, the cell cycle was analyzed using FACScalibur (BD Bioscience), and the software “FlowJo 7.6.1” was used to analyze the cell cycle results.

### Cell Migration Assay

To investigate the influence of cell proliferation on the migration, mitomycin C was used to block proliferation as the reference [33]. The migration of DPSCs was measured using Transwell plates (8 μm pore filter, Corning Costar) as the reference [35]. In brief, human DPSCs, DPSCs-vector and DPSCs-SOX2 (1×10^5^ cells) were suspended in FBS free medium, and added to the apical chambers of the insert plates (500 µL). Then 750 µL of chemoattractant (medium supplemented with 10% FBS) was added to the basal chambers. The migration assays were carried out for 24 hours at standard culture conditions. After 24 hours, the cells at the upper side of the filter (unmigrated cells) were mechanically removed, and cells that had migrated to the lower side of the filter were fixed for 30 min in 4% paraformaldehyde and further stained with crystal violet. The number of cells in six random fields was counted for each filter.

### Cell Adhesion Assay

In our study, cell adhesion assay was performed as the reference [36]. Briefly, the cells of each group were washed with PBS and harvested by trypsin, and seeded in 24-well plates (1×10^5^ cells per well) coated with fibronectin (BD Biosciences). After 1 hour of incubation at 37°C, the unattached cells were removed by washing with PBS for three times. The remaining cells were fixed with methanol, and stained with 1% crystal violet, then counted under an inverted microscope. The number of cells in six random fields was counted for each group.

### Microarray Analysis

Total RNA was extracted from DPSCs-vecctor and DPSCs-SOX2 using Trizol (Invitrogen) and the RNeasy kit (Qiagen). Samples were amplified and labeled using a NimbleGen One-Color DNA Labeling Kit. Array hybridization was analyzed with the NimbleGen Hybridization System and followed by washing with the NimbleGen wash buffer kit. The Axon GenePix 4000B microarray scanner was used for array scanning. Genes that have values greater than or equal to lower cut-off: 50.0 in 2 out of two samples were chosen for data analysis. Differentially expressed genes were identified through Fold Change filtering. Pathway Analysis and GO analysis were applied to determine the roles of these differentially expressed genes played in these biological pathways or GO terms. Finally, Hierarchical Clustering was performed to show distinguishable gene expression profiling among samples. The dataset of microarray analysis has been submitted in Gene Expression Omnibus, and the accession number is “GSE73548”.

### Knockdown of SOX2 in DPSCs-SOX2 Using siRNA

For small interfering RNA (siRNA)-mediated knockdown of SOX2, DPSCs-SOX2 were transfected with SOX2-siRNA (DPSCs-siRNA) (Santa), while the cell transfected with nontargeting siRNA were used as the control group (DPSCs-control). The detailed operation was performed according to the manufacturer’s instructions.

### Statistical Analysis

The results were expressed as means ± SEM, and statistical analysis was performed using SPSS 17.0. The differences among groups were analyzed by one-way ANOVA followed by t-test. P<0.05 was considered statistically significant.

## Results

### Characterization of DPSCs

**Fig 1:**
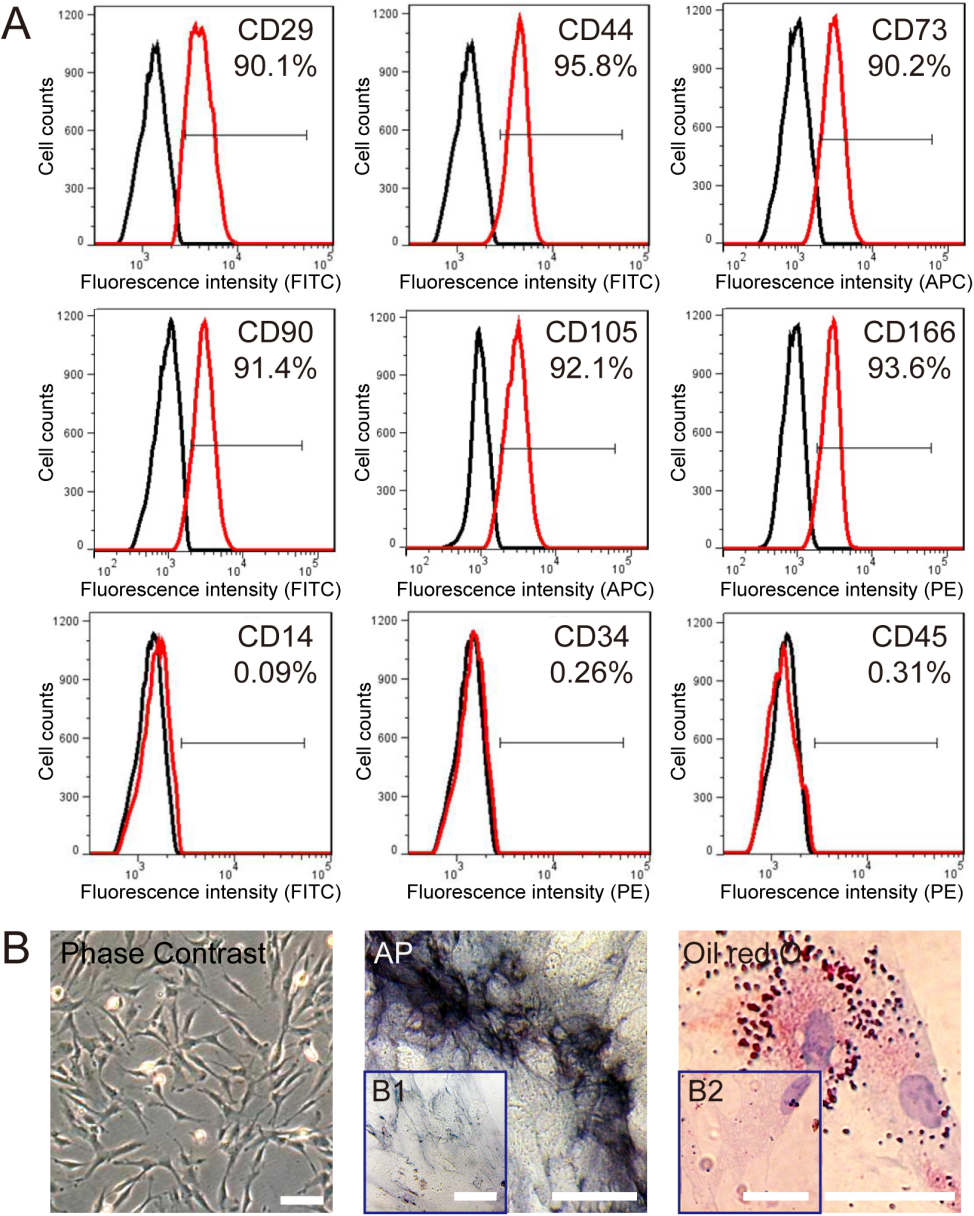
Characterization of DPSCs. (A) Immunophenotype of isolated DPSCs. Isolated DPSCs were characterized by FACS. DPSCs were positive for CD29, CD44, CD73, CD90, CD105, and CD166, and nearly negative for CD14, CD34, and CD45. (B) Differentiation characteristics of DPSCs. The phase contrast of DPSCs is shown on the left. Osteogenic differentiation was detected by AP staining (middle), and adipogenic differentiation was visualized by Oil Red O staining of the lipid vesicles (right). DPSCs cultured with normal medium were used as the negative control group for each stain (B1 and B2). The scale bar corresponds to 200 μm in Phase Contrast, and corresponds to 100 μm in AP and Oil Red O staining.

The DPSCs isolated from human dental pulp were characterized by FACS for CD14, CD29, CD34, CD44, CD45, CD73, CD90, CD105, and CD166. The results indicated that the DPSCs used in our study were positive for CD29 (90.1%), CD44 (95.8%), CD73 (90.2%), CD90 (91.4%), CD105 (92.1%), and CD166 (93.6%), and nearly negative for CD14 (0.09%), CD34 (0.26%) and CD45 (0.31%), which were usually regarded as the specific markers of hematopoietic cells (Fig 1A). In our study, the DPSCs displayed a spindle-shaped ‘‘fibroblast” appearance, and they were successfully differentiated into osteoblasts and adipocytes, as demonstrated by positive staining with alkaline phosphatase (AP) and Oil red O respectively (Fig 1B). In the negative control group, the DPSCs cultured with normal medium were used for each stain, and those cells could not be stained with AP and Oil red O kit (Fig 1B1 and 1B2).

### Analysis of DPSCs Infected with Human SOX2 Genes

**Fig 2:**
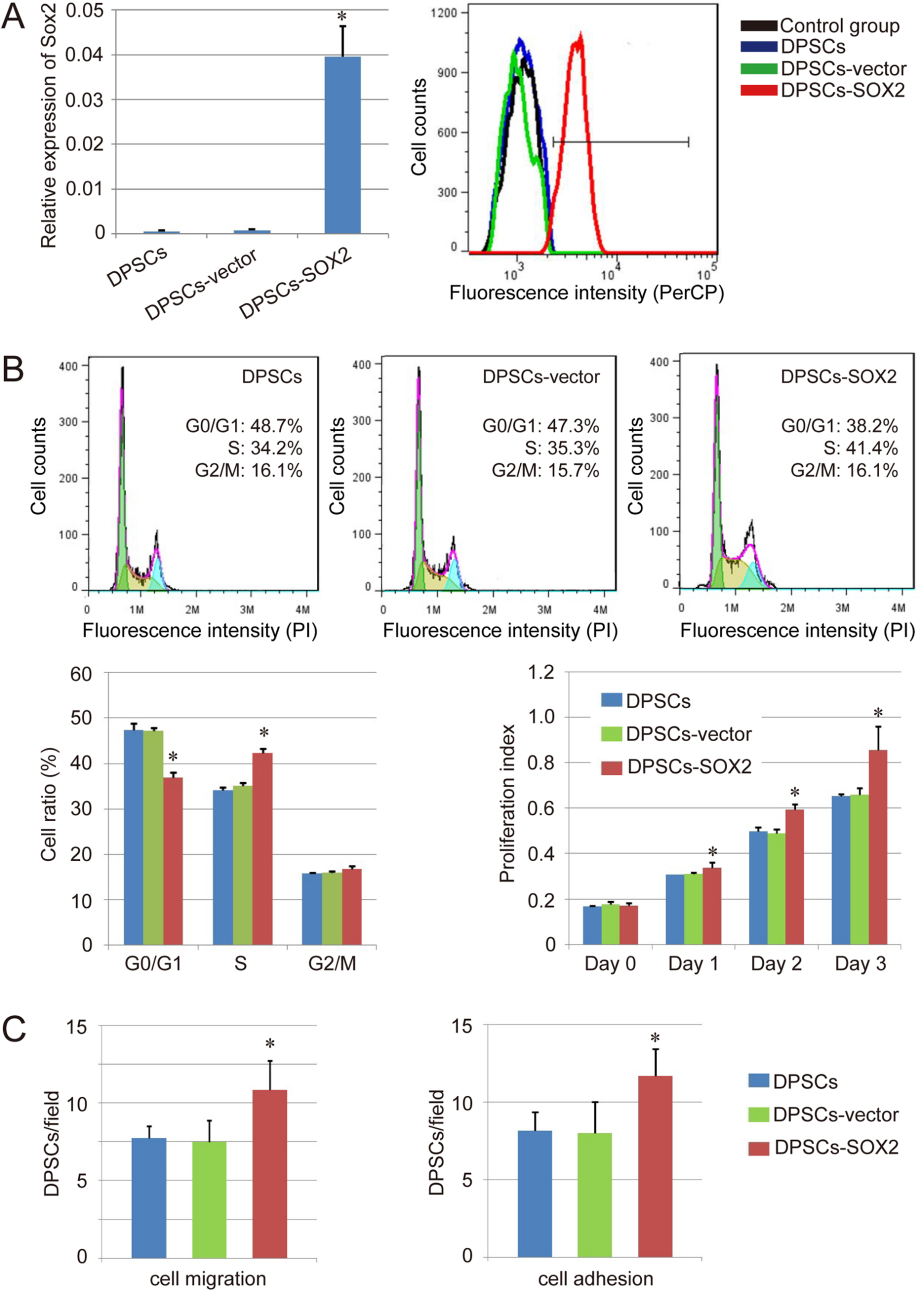
Effects of SOX2 overexpression on the biological function of DPSCs. (A) Analysis of SOX2 expression in different groups by qPCR (left) and FACS (right). (B) Effect of SOX2 overexpression on the cell proliferation. Cell cycle assay of normal DPSCs, DPSCs-vector, and DPSCs-SOX2 was performed. A representative set close to the average level of each group was shown, and followed by histograms to evaluate overall cell cycle of each group (n = 3). The colored lines in the representative indicate G0/G1 phase (green), S phase (yellow) and G2/M phase (purple) respectively. Cell proliferation index of each group was further evaluated using CCK-8. (C) Effect of SOX2 overexpression on the cell migration and adhesion ability of human DPSCs. Results are expressed as mean ± SEM. A t-test was used to compare the various groups, and P<0.05 was considered statistically significant. *P<0.05 compared with the normal DPSCs and DPSCs-vector group respectively.

To assess SOX2 expression in DPSCs infected with human SOX2 genes (DPSCs-SOX2), we performed qPCR and FACS analysis in our study. QPCR analysis indicated that the expression level of SOX2 were significantly higher in DPSCs-SOX2 compared with DPSCs and DPSCs-vector (DPSCs infected with blank vector), whereas the expression levels of SOX2 in DPSCs and DPSCs-vector were almost undetectable (Fig 2A). Besides, the FACS analysis revealed that the expression of SOX2 protein was significantly upregulated in DPSCs-SOX2 (92.8%), compared with DPSCs (0.48%) and DPSCs-vector (0.63%) (Fig 2A). These results showed that DPSCs-SOX2 were successfully generated by retroviral infection.

In addition, we evaluated the effect of SOX2 overexpression on the cell cycle of DPSCs by FACS. The results showed that in DPSCs-SOX2, 36.87±1.40% of cells were in the G0/G1 phase, 42.37±0.65% were in the S phase and 16.77±0.26% were in the G2/M phase. However, the percentage of S phase cells in DPSCs or DPSCs-vector was lower compared with DPSCs-SOX2 (34.17±0.65% v.s. 42.37±0.65%, 35.13±0.57% v.s. 42.37±0.65%, P<0.05). Besides, DPSCs-SOX2 had a reduction in the fraction of cells in G0/G1 compared with normal DPSCs and DPSCs-vector (47.36±1.40% v.s. 36.87±1.22%, 47.23±0.70% v.s. 36.87±1.22%, P<0.05). However, no significant difference existed in the G2/M phase of each group (Fig 2B).

Based on the previous finding that SOX2 overexpression improved the percentage of S phase cells, we further examined the effects of SOX2 overexpression on the proliferation ability of DPSCs with CCK-8 method. The results indicated that SOX2 overexpression in DPSCs resulted in a time-dependent increase in cell proliferation. It was apparent that the proliferation index of DPSCs-SOX2 was increased significantly compared with that of DPSCs and DPSC-vector. This data indicated that SOX2-overexpressing DPSCs hold much higher expansion potential than control cells (Fig 2B). On the other side, we evaluated the effect of SOX2 on migration and adhesion ability of DPSCs. As shown in Fig 2C, SOX2 overexpression significantly increased FBS-induced cell migration and fibronectin-induced cell adhesion compared with the control groups (P<0.05). Taken together, our results demonstrated that SOX2 overexpression could enhance the proliferation, migration and adhesion capability of DPSCs.

### Mechanism Analysis of the Effects of SOX2 Overexpression in DPSCs

**Fig 3:**
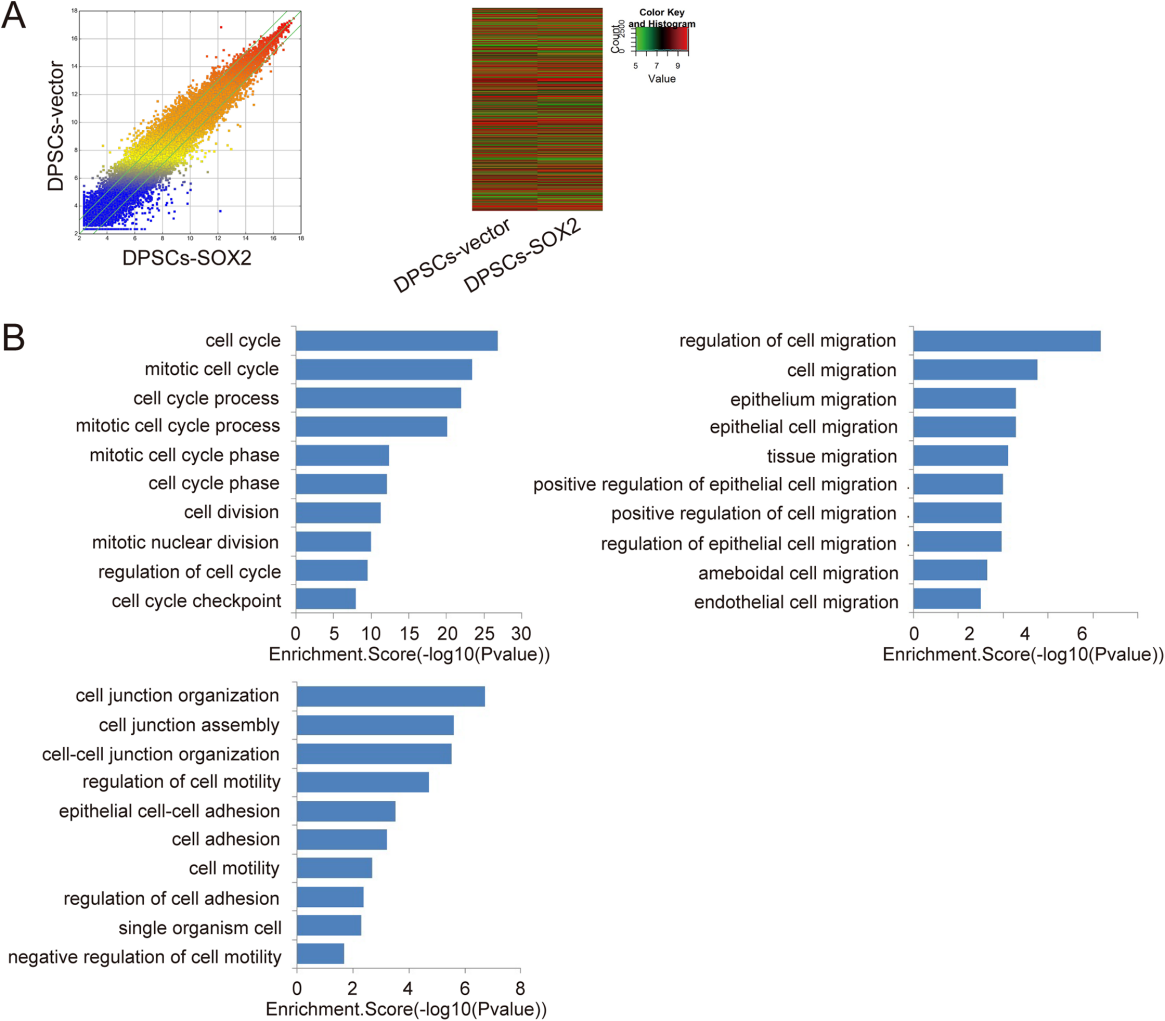
RNA microarray analysis of DPSCs-vector and DPSCs-SOX2. (A) Comparison of global gene expression profiles of DPSCs-vector and DPSCs-SOX2. The values of X and Y axes in the Scatter-Plot were normalized signal values of each sample (log2 scaled). The green lines are Fold Change Lines (The default fold change value given was 2.0). Over two fold alterations of genes between two compared samples could be found above the top green line and below the bottom green line. In Hierarchical Clustering for ‘‘all targets value”, ‘‘Red” indicates high relative expression, and ‘‘green” indicates low relative expression. (B) Analysis of Gene Ontology project associated with cell proliferation, cell migration and cell adhesion. The bar plots showed the top ten Enrichment Score value of the significant enrichment terms.

Gene expression in both DPSCs-SOX2 and DPSCs-vector was verified by RNA arrays (Fig 3). The Scatter-Plot and Hierarchical Clustering were performed based on all target values to assess the difference in gene expression between the DPSCs-SOX2 and DPSCs-vector, and the results showed distinguishable gene expression profiling between the two groups (Fig 3A). The Gene Ontology project provided a controlled vocabulary to describe genes, associated with cell cycle and cell adhesion or junction, upregulated in DPSCs-SOX2 compared with DPSCs-vector. The bar plots showed the top ten Enrichment Score value of the significant enrichment terms associated with cell cycles, cell migration and cell adhesion or junction respectively (Fig 3B).

The pathways associated with cell cycle, cell migration and cell adhesion were further analyzed through RNA arrays in our study. The result showed that some genes in both cell cycle pathway and DAN replication pathway were upregulated in DPSCs-SOX2 compared with DPSCs-vector, indicating that SOX2 may regulate cell proliferation and cell cycle though the two pathways (S1 Fig). In recent years, PI3K-AKT signaling pathway has been demonstrated to regulate mesenchymal stem cell migration [37–39]. In our study, we also found that the some genes in PI3K-AKT signaling pathway were up-regulated in DPSCs-SOX2 compared with DPSCs-vector. This result might lead to the enhancement of cell migration ability (S2 Fig). Besides, we found genes associated with cell adhesion were upregulated in DPSCs-SOX2, including genes in focal adhesion pathway, adherens junction pathway and cell adhesion molecules pathway (S3 Fig). The enhancement of those genes expression may lead to the increase of cell adhesion ability.

**Fig 4:**
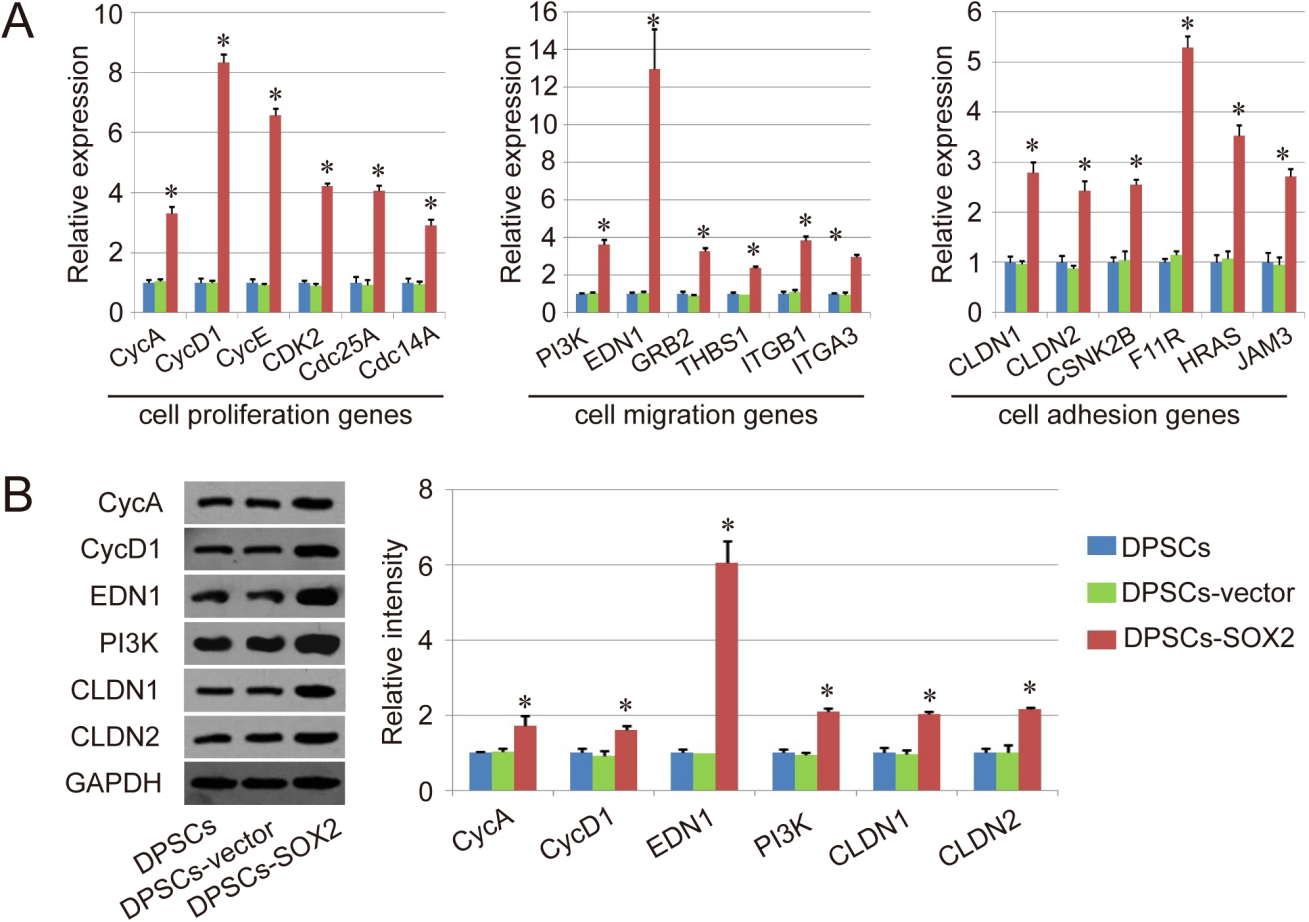
Detection of the key genes about cell cycle, cell migration and cell adhesion with qPCR and western-blot. (A) QPCR detection of the key genes about cell cycle (CycA, CycD1, CycE, CDK2, Cdc25A and Cdc14A), cell migration (PI3K, EDN1, GRB2, THBS1, ITGB1 and ITGA3) and cell adhesion (CLDN1, CLDN2, CSNK2B, F11R, HRAS and JAM3). (B) Detection of the key genes about cell cycle (CycA and CycD1), cell migration (PI3K and EDN1) and cell adhesion (CLDN1 and CLDN2) with western-blot. A representative set close to the average level of each group was shown, and the original blots have been provided in S4 Fig Similar results were obtained in three independent experiments. Results are expressed as mean ± SEM. A t-test was used to compare the various groups, and P<0.05 was considered statistically significant. *P<0.05 compared with the normal DPSCs and DPSCs-vector group respectively.

To further confirm the results of RNA array, qPCR and western-blot analysis were performed in our study. At the level of mRNA, some key genes about cell cycle (CycA, CycD1, CycE, CDK2, Cdc25A and Cdc14A), cell migration (PI3K, EDN1, GRB2, THBS1, ITGB1 and ITGA3) and cell adhesion (CLDN1, CLDN2, CSNK2B, F11R, HRAS and JAM3) were upregulated in DPSCs-SOX2, compared with DPSCs and DPSCs-vector (P<0.05), and there are no significant difference between DPSCs and DPSCs-vector (Fig 4A). The changes in the protein levels of CycA, CycD1, PI3K1, EDN1, CLDN1 and CLDN2 were further detected with western-blot, and the result was similar to that of qPCR, showing the upregulation of those genes expression in the group of DPSCs-SOX2, compared with DPSCs and DPSCs-vector (P<0.05), and no significant difference between the group of DPSCs and DPSCs-vector (Fig 4B).

Therefore, the mechanism analysis indicated that SOX2 overexpression could improve cell proliferation ability of DPSCs mainly through the upregulation of key genes in cell cycle pathway and DAN replication pathway. Besides, it also could upregulate some key genes associated with cell migration, adhesion and junction, further result in the increase of cell migration and adhesion ability of DPSCs.

### Effect of SOX2-siRNA on the Cell Biological Ability of DPSC-SOX2

**Fig 5:**
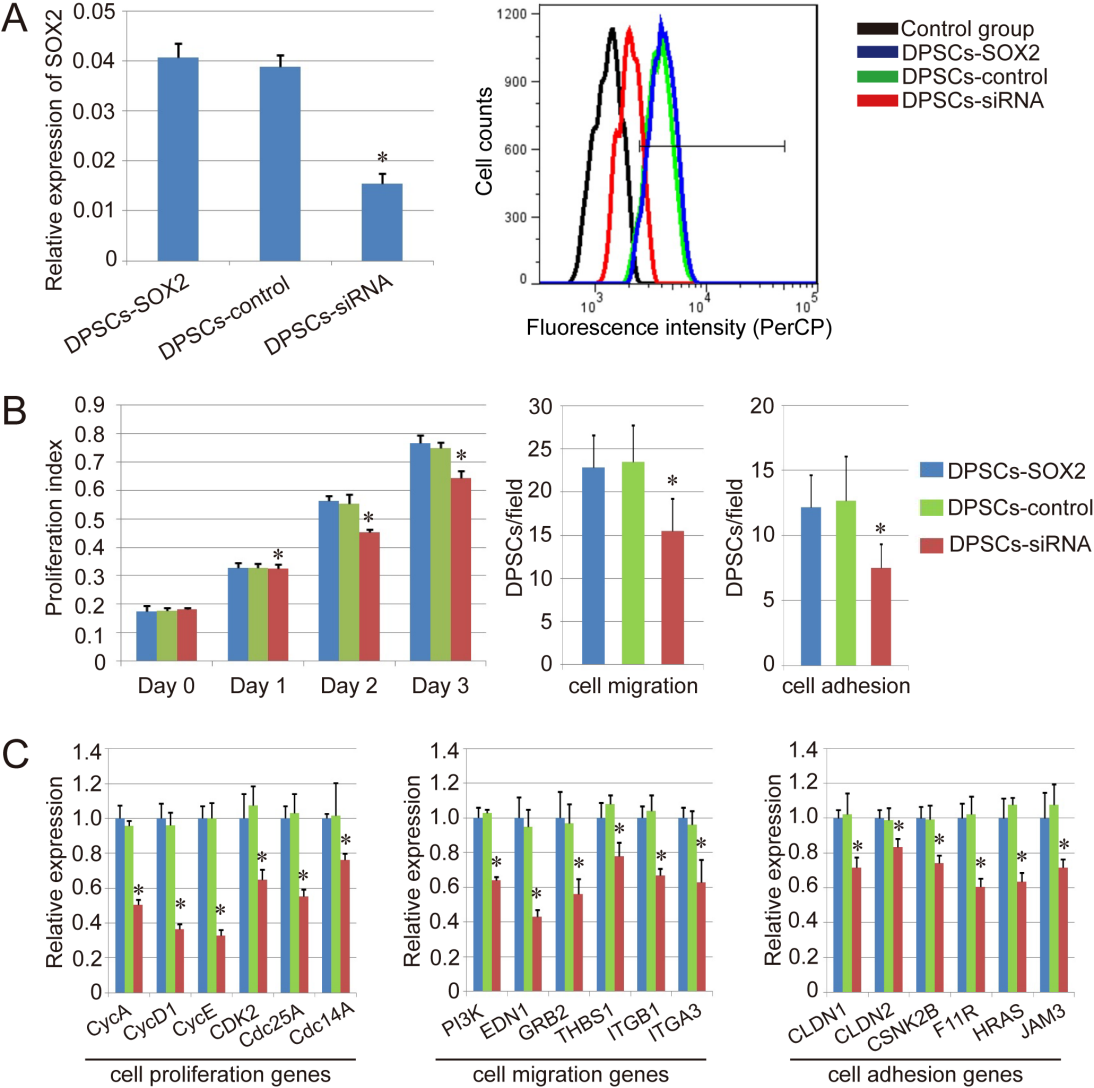
Effect of SOX2-siRNA on the biological ability of DPSC-SOX2. (A) Analysis of effect of SOX2-siRNA on SOX2 expression in different groups by qPCR (left) and FACS (right). (B) Effect of SOX2-siRNA on the cell proliferation, migration and adhesion ability of DPSCs-SOX2. (C) QPCR detection of the key genes about cell cycle, cell migration and cell adhesion in DPSCs-SOX2, DPSCs-control and DPSCs-siRNA. Similar results were obtained in three independent experiments. Results are expressed as mean ± SEM. A t-test was used to compare the various groups, and P<0.05 was considered statistically significant. *P<0.05 compared with the normal DPSCs-SOX2 and DPSCs-control group respectively.

To further confirm the biological function of SOX2 overexpression in DPSCs, a rescue experiment was performed with SOX2-siRNA in DPSC-SOX2. Herein, qPCR and FACS analysis were also performed to assess SOX2 expression in DPSCs-SOX2 treated with SOX2-siRNA (DPSCs-siRNA). The result of qPCR analysis indicated that the expression of SOX2 was significantly downregulated in DPSCs-siRNA compared with DPSCs-SOX2 and DPSCs-SOX2 treated with nontargeting control siRNA (DPSCs-control) (Fig 5A). Besides, the FACS results showed that the expression level of SOX2 protein was significantly lower in DPSCs-siRNA (28.5%), compared with DPSCs-SOX2 (90.6%) and DPSCs-control (89.2%) (Fig 5A). These results indicated that the SOX2-siRNA was effective in our study.

The cell proliferation ability, cell migration ability as well as cell adhesion ability of DPSCs-siRNA, were further evaluated. As expected, the results showed that the proliferation ability, migration ability and the adhesion ability was inhibited in DPSCs-siRNA, compared with DPSCs-SOX2 and DPSCs-control, and those biological abilities of DPSCs-SOX2 and DPSCs-control did not show significant difference (Fig 5B). We also detected the changing of genes associated with cell cycle, cell migration and cell adhesion with qPCR as before, and the results showed the downregulated expression of those genes about cell cycle (CycA, CycD1, CycE, CDK2, Cdc25A and Cdc14A), cell migration (PI3K, EDN1, GRB2, THBS1, ITGB1 and ITGA3) and cell adhesion (CLDN1, CLDN2, CSNK2B, F11R, HRAS and JAM3) in different degree (P<0.05), indicating the effect of SOX2 on the promotion of cell proliferation, cell migration as well as cell adhesion in DPSCs (Fig 5C).

## Discussion

As a transcription factor involved in the determination of cell fate and in the regulation of stem cell development, SOX2 has been investigated by scientists for years. Several groups have demonstrated that SOX2 holds the potential to improve cell proliferation through regulating cell cycle [16, 40–42]. Our results further confirmed this conclusion in human DPSCs, and developed a novel method to enhance the proliferation ability and lifespan of the stem cells.

For some kinds of cancer cells, SOX2 has been demonstrated to hold the potential to promote cell migration and invasion [30, 43, 44]. In our study, we also found the expression of some genes associated with cell migration upregulated through RNA array, and further functional detections confirmed that the similar function of SOX2 on cell migration indeed exist in human DPSCs. Some scientists have indicated that mesenchymal stem cells were largely localized in pulmonary capillaries after intravenous administration for disease treatment, and the development of novel strategies in enhancing cell migration to target tissues is regarded as a prerequisite for the success of stem cell-based systemic therapies [33, 45]. However, whether our gene modified DPSCs hold better potential to migrate to damaged tissue or organ, still need our further investigation in vivo.

In addition, few investigations about the effect of SOX2 on cell adhesion have been reported so far. Recently, Galle et al found that EDTA conditioning of dentine could promote the adhesion and migration of DPSCs towards or onto dentine, and the pretreatment with EDTA as the final step of an irrigation protocol for regenerative endodontic procedures hold the potential to act favorably on new tissue formation within the root canal [46]. Their study established a novel method to enhance cell adhesion ability through the pretreatment of dentine material. Differently, we found that the overexpression of SOX2 could increase the expression of some genes about cell adhesion, and further enhance the adhesive ability of DPSCs. This result provides a new insight into the establishment of novel seed cells with strong adhesive ability in tissue engineering, in which the novel seed cells can attach to scaffold easily and further develop into functional tissue or organ under appropriate regulation. However, the feasibility of this idea still needs detection, and whether the similar function on cell adhesion of SOX2 exists in other kinds of cells, also needs our further exploration.

Even though SOX2 has been demonstrated to hold the potential to promote DPSCs proliferation, migration and adhesion ability through cell cycle pathway, PI3K-AKT pathway and some cell adhesion pathways, we are still unclear which genes are regulated by SOX2 directly in those pathways, and those genes should be the key points for us to understand the biological function of SOX2 intensively. Therefore, maybe the detailed analysis should be performed for each pathway associated with SOX2 overexpression, and the enhanced understanding of the regulating mechanism of SOX2 in different kinds of cells, may help us to develop the novel stem cell source for regenerative medicine with gene regulation.

## Conclusion

In conclusion, we for the first time investigated the effects of SOX2 on proliferation, migration and adhesion of DPSCs and found that SOX2 overexpression could enhance proliferation, migration and adhesion capacity of DPSCs. We also observed that the overexpression of SOX2 could upregulate some genes about cell proliferation, migration and adhesion, and further activate the associated signal pathways. More importantly, application of SOX2-siRNA could significantly inhibit SOX2-induced DPSCs proliferation, migration and adhesion, as well as expression of cell cycle genes, migration regulators and adhesive molecules. These findings provided a novel strategy to develop seed cells with strong proliferation, migration and adhesion ability for tissue engineer through SOX2 overexpression.

## Supporting Information

S1 Fig Gene analysis in cell cycle pathway and DAN replication pathway through RNA microarray. “Yellow” indicated the upregulated genes, and “cyan” indicated the genes without significant changing.
(PDF)

S2 Fig Gene analysis in PI3K-AKT signaling pathway through RNA microarray. “Yellow” indicated the upregulated genes, and “cyan” indicated the genes without significant changing.
(PDF)

S3 Fig Gene analysis in focal adhesion pathway, adherens junction pathway and cell adhesion molecules pathway through RNA microarray. “Yellow” indicated the upregulated genes, and “cyan” indicated the genes without significant changing.
(PDF)

S4 Fig The original blots of the representative in Fig 4. (PDF)
