# Supplementary figures and images for: Effects of SOX2 on Proliferation, Migration and Adhesion of Human Dental Pulp Stem Cells
Source: PLoS One. 2015 Oct 23;10(10):e0141346. doi: 10.1371/journal.pone.0141346 (PMC4619695; doi:10.1371/journal.pone.0141346)

# S2 Fig.

## PI3K-AKT signaling pathway

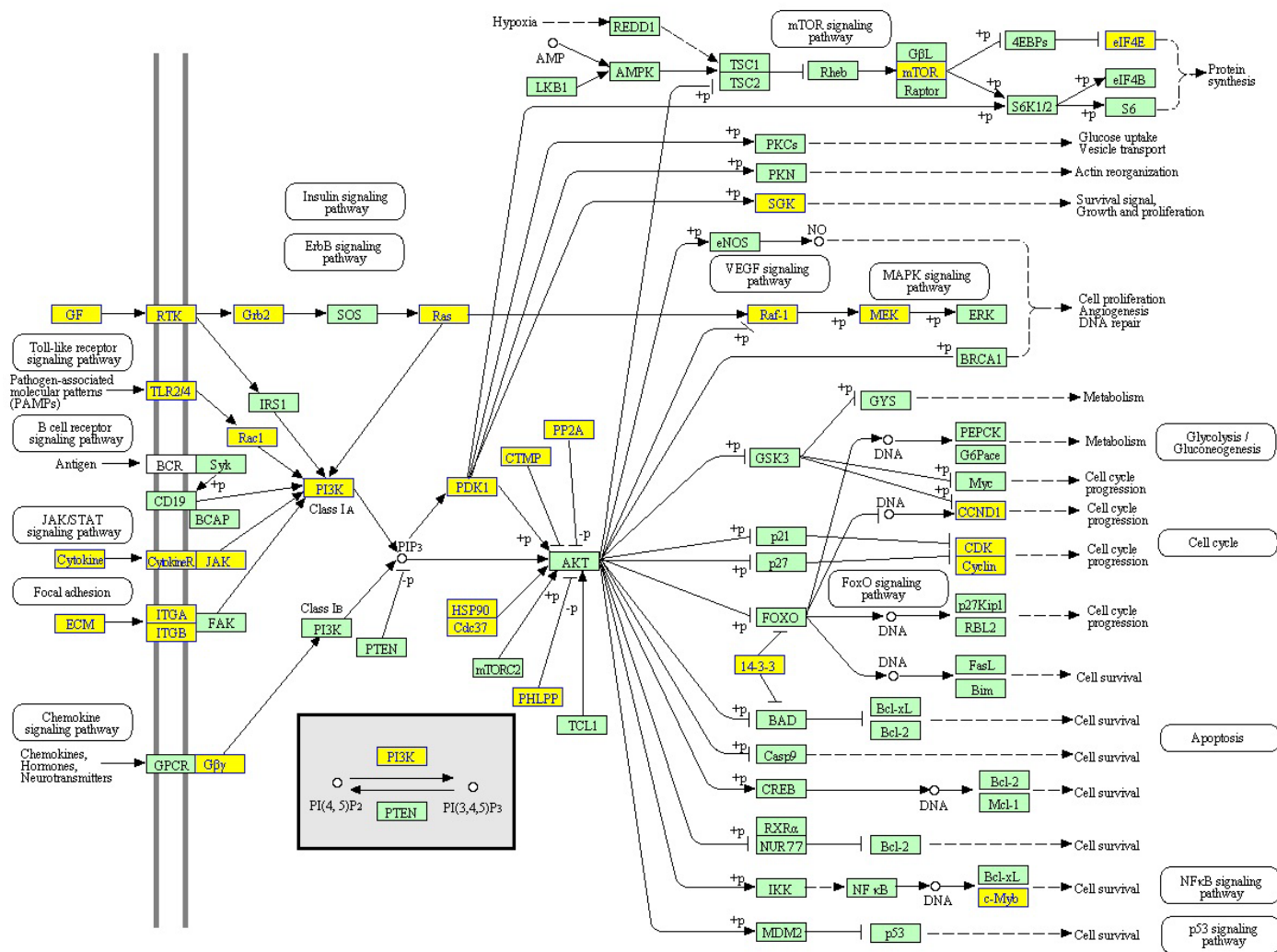

Supplement: S2 Fig — “Yellow” indicated the upregulated genes, and “cyan” indicated the genes without significant changing. (PDF) [file pone.0141346.s002.pdf]

S3 Fig. 3

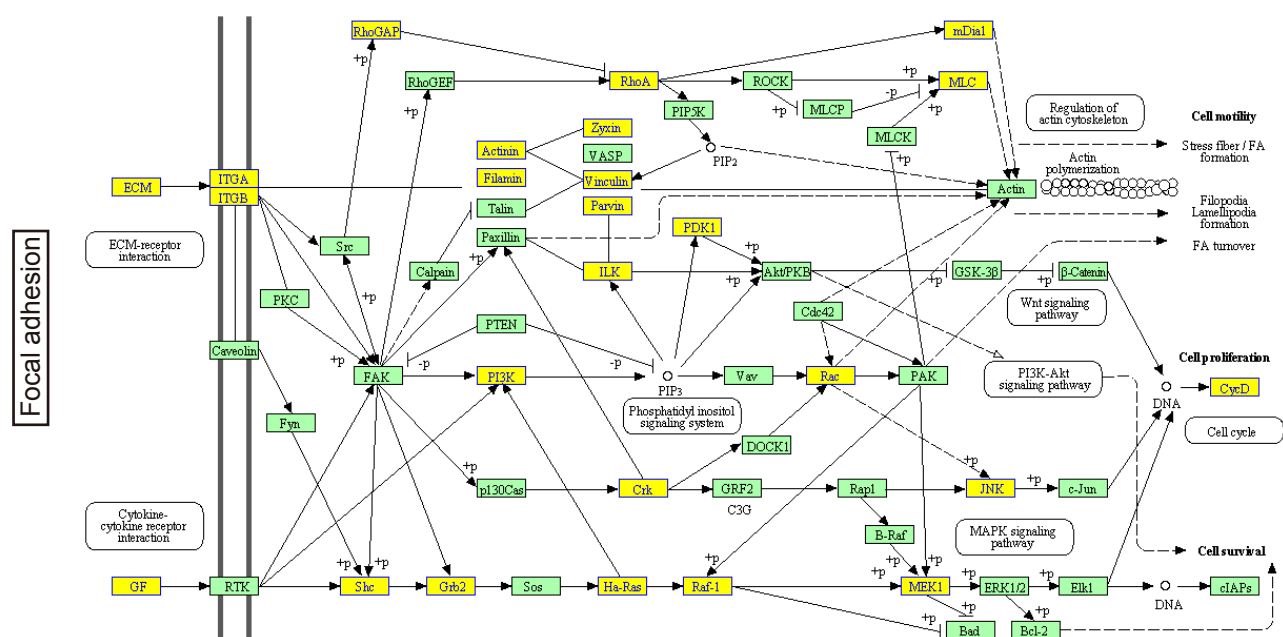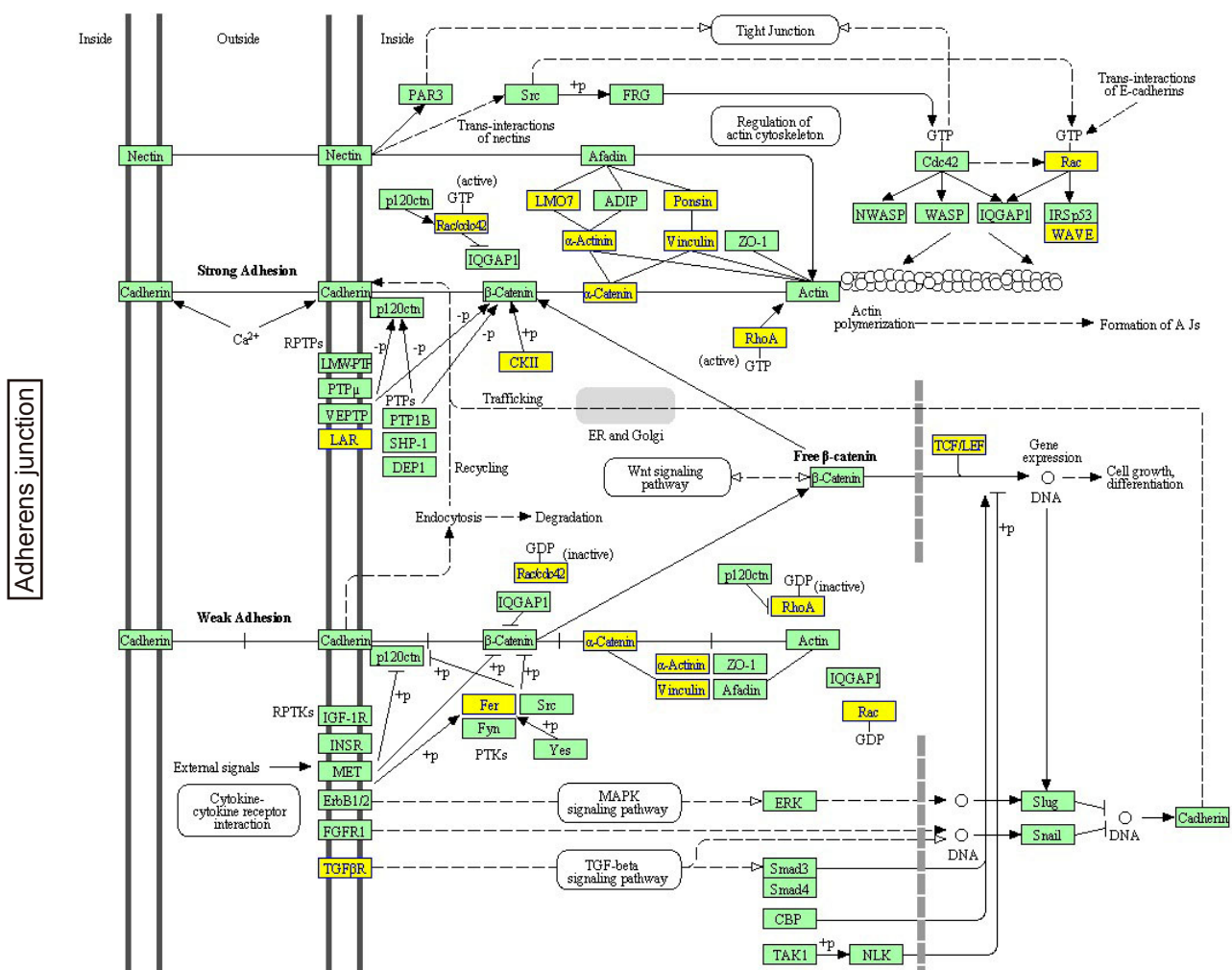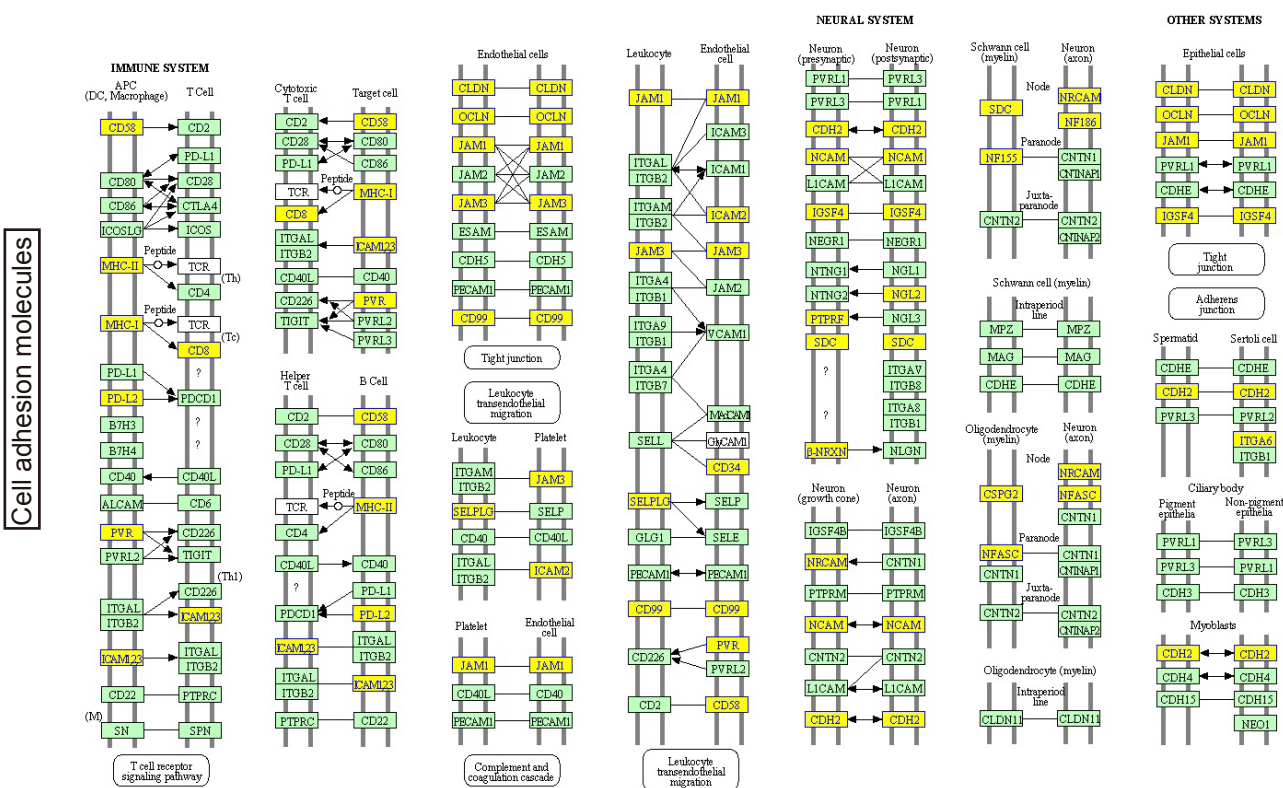

Supplement: S3 Fig — “Yellow” indicated the upregulated genes, and “cyan” indicated the genes without significant changing. (PDF) [file pone.0141346.s003.pdf]

S4 Fig.

CycA

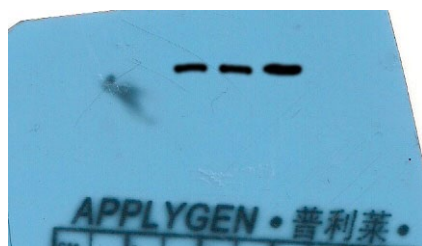

CycD

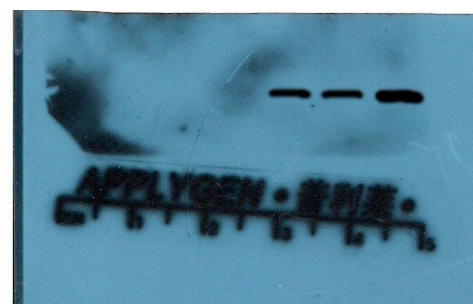

EDN1

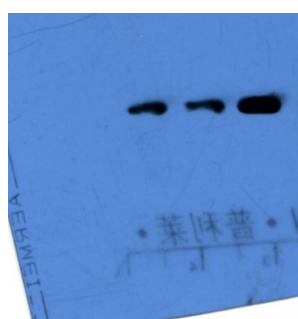

PI3K

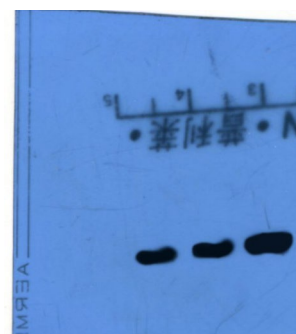

CLDN1

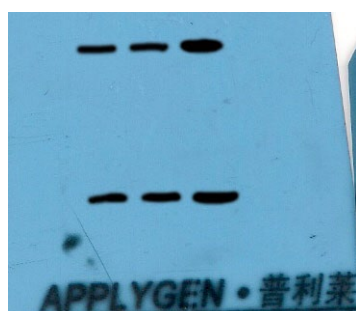

CLDN2

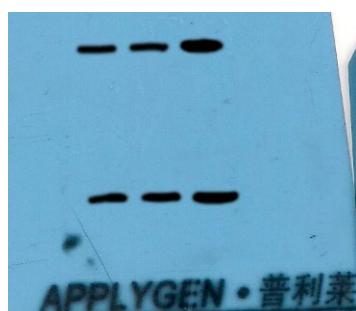

GAPDH

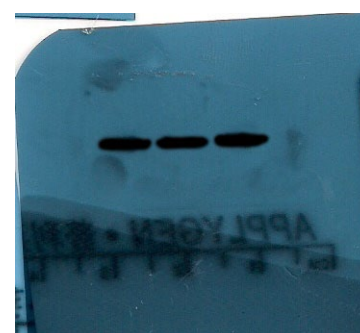

Supplement: S4 Fig — (PDF) [file pone.0141346.s004.pdf]
