# Supplementary material for: Effects of SOX2 on Proliferation, Migration and Adhesion of Human Dental Pulp Stem Cells
Source: PLoS One. 2015 Oct 23;10(10):e0141346. doi: 10.1371/journal.pone.0141346 (PMC4619695; doi:10.1371/journal.pone.0141346)

S1 Fig.

Cell cycle

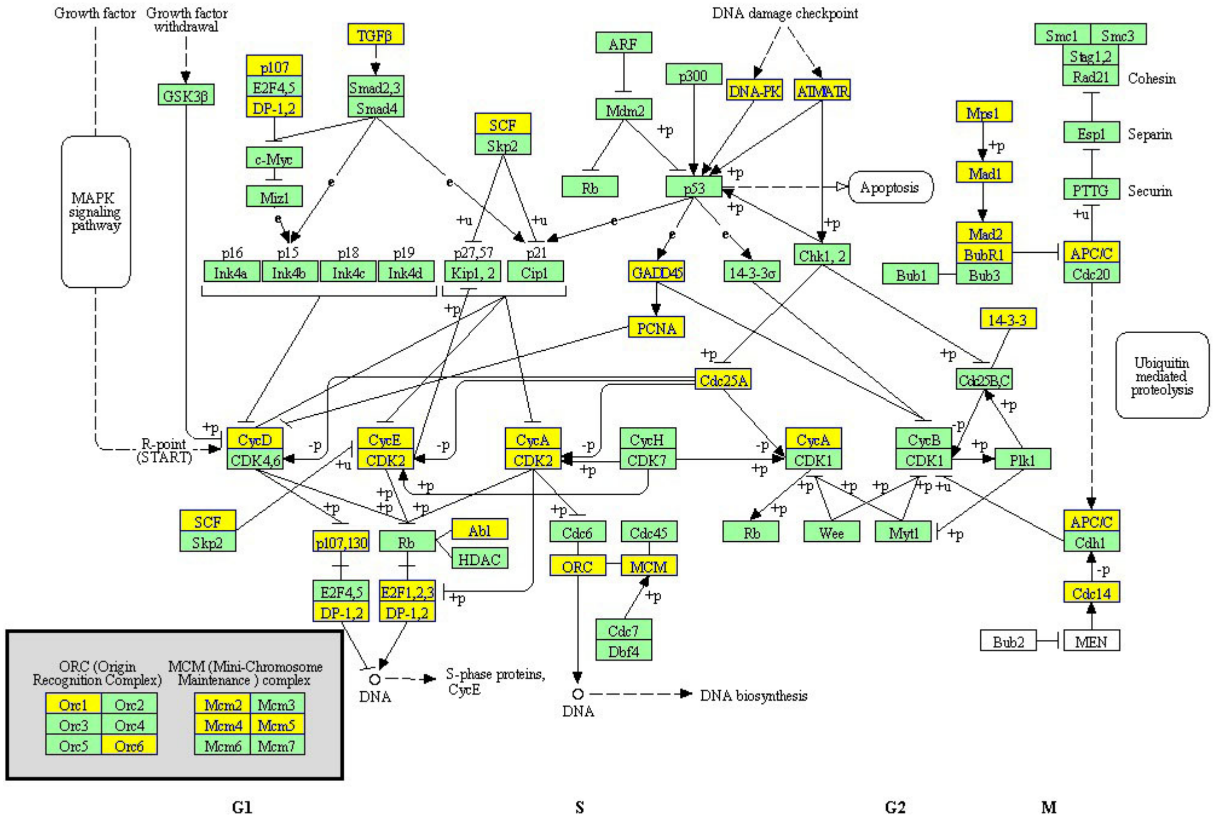

DAN replication

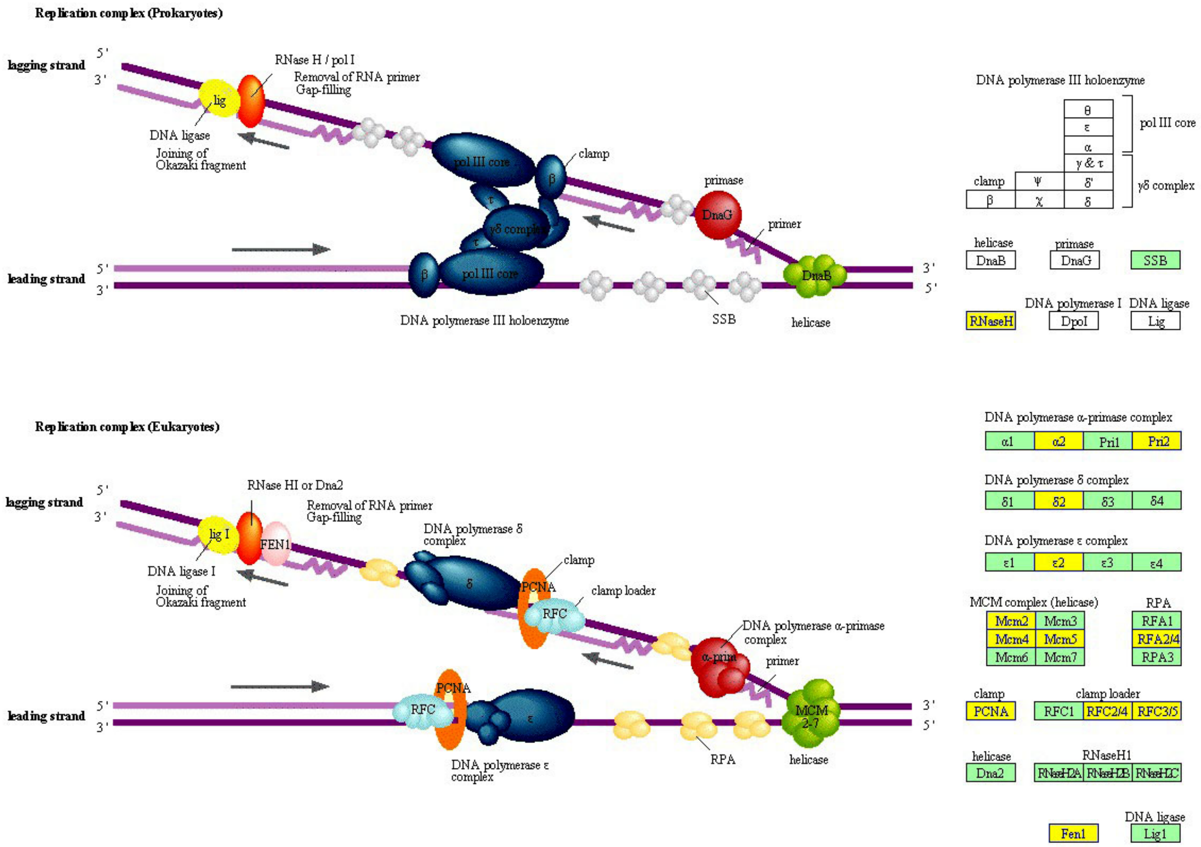

Supplement: S1 Fig — “Yellow” indicated the upregulated genes, and “cyan” indicated the genes without significant changing. (PDF) [file pone.0141346.s001.pdf]
